# A prediction nomogram for moderate-to-severe bronchopulmonary dysplasia in preterm infants < 32 weeks of gestation: A multicenter retrospective study

**DOI:** 10.3389/fped.2023.1102878

**Published:** 2023-04-03

**Authors:** Jing Zhang, Kai Mu, Lihua Wei, Chunyan Fan, Rui Zhang, Lingling Wang

**Affiliations:** ^1^ Department of Pediatric, Department of Pediatrics, The First Affiliated Hospital of Shandong First Medical University & Shandong Provincial Qianfoshan Hospital, Shandong Engineering and Technology Research Center for Pediatric Drug Development, Jinan, China; ^2^ Department of Neonatology, Affiliated Hospital of Jining Medical College, Jining, China; ^3^ Department of Pediatrics, Zibo First Hospital, Zibo, China

**Keywords:** bronchopulmonar dysplasia, preterm, nomogram, prediction model, multi-center study, retrospective study

## Abstract

**Background:**

Moderate-to-severe bronchopulmonary dysplasia (msBPD) is a serious complication in preterm infants. We aimed to develop a dynamic nomogram for early prediction of msBPD using perinatal factors in preterm infants born at <32 weeks' gestation.

**Methods:**

This multicenter retrospective study conducted at three hospitals in China between January 2017 and December 2021 included data on preterm infants with gestational age (GA) < 32 weeks. All infants were randomly divided into training and validation cohorts (3:1 ratio). Variables were selected by Lasso regression. Multivariate logistic regression was used to build a dynamic nomogram to predict msBPD. The discrimination was verified by receiver operating characteristic curves. Hosmer-Lemeshow test and decision curve analysis (DCA) were used for evaluating calibration and clinical applicability.

**Results:**

A total of 2,067 preterm infants. GA, Apgar 5-min score, small for gestational age (SGA), early onset sepsis, and duration of invasive ventilation were predictors for msBPD by Lasso regression. The area under the curve was 0.894 (95% CI 0.869–0.919) and 0.893 (95% CI 0.855–0.931) in training and validation cohorts. The Hosmer−Lemeshow test calculated *P* value of 0.059 showing a good fit of the nomogram. The DCA demonstrated significantly clinical benefit of the model in both cohorts. A dynamic nomogram predicting msBPD by perinatal days within postnatal day 7 is available at https://sdxxbxzz.shinyapps.io/BPDpredict/.

**Conclusion:**

We assessed the perinatal predictors of msBPD in preterm infants with GA < 32 weeks and built a dynamic nomogram for early risk prediction, providing clinicians a visual tool for early identification of msBPD.

## Introduction

Bronchopulmonary dysplasia (BPD) is one of the most common and serious complications in very preterm neonates. Infants with moderate-to-severe BPD (msBPD) are more likely to die, and those who survive have an increased risk of asthma, chronic lung disease in adulthood, neurodevelopmental impairment, and growth failure (1). However, there is no definite effective therapies for the prevention and treatment of msBPD.

Although management strategies for very premature infants have greatly progressed, studies have shown that BPD affects 10%–89% of very preterm infants (2, 3). Many studies have shown that perinatal factors were risk factors for developing msBPD (4–6). As the immature lung of very preterm infants is the most vulnerable period during the first week of life. Perinatal prediction may help clinicians perform interventions for msBPD as early as possible. Laughon et al. (7) firstly reported prediction model for BPD and death in preterm with 23–30 weeks' GA. Greenberg et al. (8) updated a web based estimator for BPD and death in preterm with GA ranging from 23 + 0/7 weeks to 28 + 6/7 weeks. Min Song et al. (5) developed a nomogram for msBPD in a single-center study, which included the N-terminal Pro-B-Natriuretic Peptide level, gestational age (GA) and ventilation-assisted ventilation. Amit Sharma et al. (9) reported early prediction of msBPD for extremely premature infants. A meta-analysis conducted by Onland et al. reported poor predictability in most established prediction models for BPD (10). Although many prediction models for have been developed using logistic regression, no machine learning model for predicting msBPD in Chinese multi-center populations.

A multi-center study was conducted to establish a dynamic nomogram using least absolute shrinkage and selection operator (Lasso) regression for predicting msBPD. The calibration of the nomogram was assessed using Hosmer–Lemeshow test. A dynamic diagnosis model nomogram was a simple statistical visual tool which has been widely used. The occurrence of msBPD is multifactorial and related to both intrauterine factors and postnatal clinical management; we selected perinatal risk factors for the analysis by Lasso regression. Hence, our study aimed to develop a dynamic nomogram for the early prediction of msBPD to improve predictive validity and provide the evidence for clinicians to prevent msBPD.

## Methods

### Study design and population

All data of this multi-center retrospective study were obtained from three hospitals including the First Affiliated Hospital of Shandong First Medical University, Affiliated Hospital of Jining Medical College, and the First Hospital of Zibo, between January 2017 and December 2021. All three hospitals underwent standardized training before the start of the project. This study was approved by the ethics review board of the First Affiliated Hospital of Shandong First Medical University [XMSBLL2021(425)] and was recognized by all participating hospitals. A waiver of consent was granted at all sites, owing to the use of unidentified patient data.

The included infants were randomly assigned in a 3:1 ratio dividing into the training and validation cohorts. The inclusion criteria were as follows: (1) the preterm infants born at <32 weeks’ gestation and >23 weeks' gestation (2) hospitalization for ≥28 days in the three hospitals. Infants with GA with 29 + 0/7 to 31 + 6/7 weeks were included, due to the incidence of BPD, which was 19.3% in Chinese preterm cohort study (3). The exclusion criteria were as follows: multiple malformations. The data on infants readmitted or transferred between hospitals in the collaborative network adopted a unique identification. All included infants were Han Chinese.

### Definitions

The diagnosis and grading of BPD were based on the National Institutes of Child and Human Development (NICHD) 2001 (11). BPD is diagnosed when preterm infants require supplemental oxygen (fraction of inspired oxygen, FiO_2_ > 21%) for 28 days. Mild BPD id diagnosed when infants do not require FiO_2_ at postmenstrual age (PMA) 36 weeks; moderate BPD as receiving FiO_2 _< 30% and severe BPD as receiving FiO_2 _≥ 30% or positive pressure at PMA 36 weeks. SGA was defined as birth weight ≤ the 10th percentile for the same GA. Early onset sepsis (EOS) was diagnosed in preterm infants with bacteremia or bacterial meningitis ≤ 72 h after birth (12). The duration of invasive ventilation was calculated as the number of days of invasive ventilation within 7 days after birth.

### Data collection

Parental factors (maternal age, medication use during pregnancy, smoking and antenatal corticosteroid), risk factors before delivery (gestational diabetes mellitus, intrahepatic cholestasis of pregnancy, hypertensive disorders of pregnancy, chorioamnionitis, premature rupture of membrane and infectious disease within 7 days before delivery), basic information of infants (GA, birth weight, Apgar score at 1 and 5 min and mode of delivery), disease diagnostic before 7 DOL (EOS and hemodynamically significant patent ductus arteriosus) and treatment during the first 7 DOL (pulmonary surfactant, caffeine, invasive mechanical ventilation, and parenteral nutrition) were collected.

### Development and assessment of the nomogram

Multivariate logistic regression was used to build a nomogram model to predict the occurrence of msBPD and plot the receiver operating characteristic (ROC) curves. The areas under the ROC curve (AUC) and concordance index (C-index) were calculated to measure the predictive ability and accuracy of the model. The goodness of fit was calibrated by using a calibration curve and Hosmer–Lemeshow test. The utility of the model for decision making was evaluated using decision curve analysis (DCA). DCA is critical method for assessing the clinical utility of clinical predictive models (13), and can address the limitations of ROC curve (14). Each variable corresponds to a point on the axis of the nomogram, and the corresponding score of the variable was obtained. The sum score of each variable was obtained, the total score corresponded to the point on the risk axis, and the risk value of msBPD was obtained.

### Statistical analysis

The sample size calculation was performed using PASS version 11.0.7 (NCSS Statistical Software, Kaysville, UT, USA). The number of events per candidate variable was restricted to >20. The Lasso regression method performs variable selection as an alternative to the subset selection method to reduce model complexity. This method can prevent the model from overfitting and avoids the need for multiple test corrections (15). Normally distributed data are presented as mean ± standard deviation, and nonnormally distributed data are presented as medians and interquartile ranges. Categorical variables are expressed as frequencies and percentages. The demographic variables were summarized using descriptive statistics. Differences between the groups were compared using the chi-square or Fisher's tests. All statistical tests were two-sided. Statistical significance was set at *P *< 0.05. All analyses and a dynamic nomogram were used to visualize the model, which was achieved using R software (version4.0.4; R Foundation for Statistical Computing, Vienna, Austria).

## Results

### General characteristics

**Figure 1:**
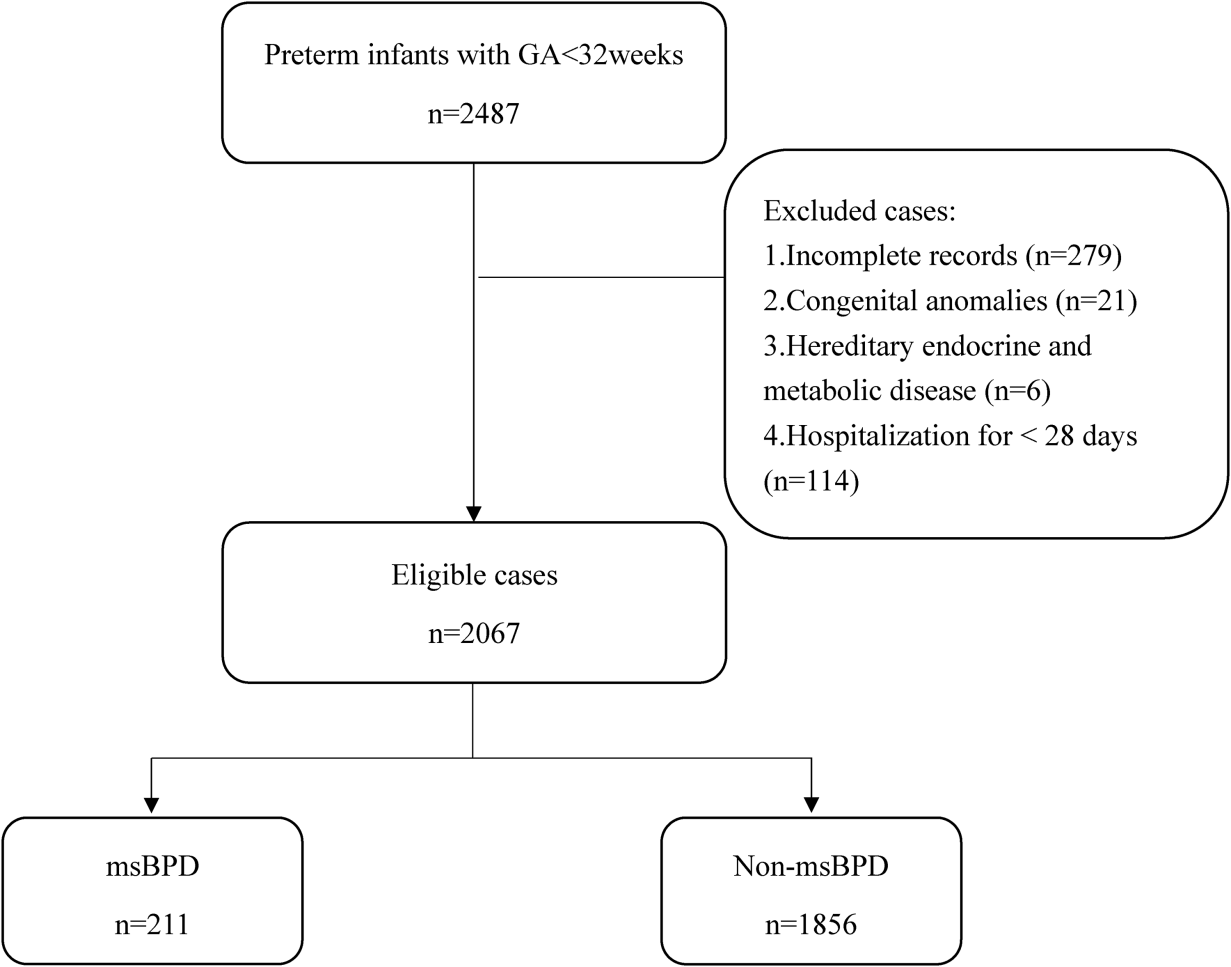
Flowchart for patient selection. GA, gestational age; msBPD, moderate-to-severe BPD.

A total of 2,373 premature infants who met the inclusion criteria in the three hospitals between January 2017 and December 2021. 279 infants were excluded because of incomplete records. 2,067 patients were included in our study (Figure 1). The data was randomly divided into training (1,551 cases) and validation cohorts (516 cases) according to a 3:1 ratio. In the training cohort, the clinical information of 1,551 patients were obtained. The general characteristics including GA, birth weight, and sex, showed no statistical differences in the two cohort. Data from the training cohort were used to construct the dynamic nomogram. According to the NICHD 2001 grading of BPD (11), 331, 150, and 61 infants were diagnosed with mild, moderate, and severe BPD, respectively. The baseline characteristics of the training and validation sets are listed in Table 1.

**Table 1:** Baseline characteristics of all patients in the training cohort and validation cohort.

Variables		Training cohort (*n* = 1551)	Validation cohort (*n* = 516)	*P*-value
GA (weeks)		29.7 ± 1.6	29.6 ± 1.7	0.45
Birth weight (kilograms)		1.33 ± 0.34	1.33 ± 0.34	0.52
Gender	Male	850 (54.8%)	269 (52.1%)	0.29
	Female	701 (45.2%)	247 (47.9%)	
Apgar5-min score		8 (7,9)	8 (7,9)	0.43
Mode of delivery	Caesarean delivery	417 (26.9%)	130 (25.2%)	0.45
	Vaginal delivery	1,134 (73.1%)	386 (74.8%)	
PROM	+	493 (31.8%)	156 (30.2%)	0.76
	−	1,058 (68.2%)	360 (69.8%)	
ICP	+	124 (8.0%)	48 (9.3%)	0.12
	−	1,427 (92.0%)	468 (90.7%)	
GDM	+	244 (15.7%)	77 (14.9%)	0.66
	−	1,307 (84.3%)	439 (85.1%)	
HDP	+	367 (23.7%)	118 (22.9%)	0.71
	−	1,184 (76.3%)	398 (77.1%)	
Prenatal steroid	+	1,057 (68.1%)	329 (63.8%)	0.07
	−	494 (31.9%)	187 (36.2%)	
SGA	+	120 (7.7%)	53 (10.3%)	0.07
	−	1,431 (92.3%)	463 (89.7%)	
EOS	+	109 (7.0%)	51 (9.9%)	0.03
	−	1,442 (93.0%)	465 (90.1%)	
PDA	+	936 (60.3%)	342 (66.3%)	0.02
	−	615 (39.7%)	174 (33.7%)	
PS	+	427 (27.5%)	147 (28.5%)	0.67
Duration of invasive ventilation (day)		0 (0,5)	0 (0,5)	0.61
msBPD	+	171 (11.0%)	40(7.8%)	0.03
	−	1380(89.0%)	476(92.3%)	

+, yes; -, no; GA, gestational age; PROM, premature rupture of membrane; ICP, intrahepatic cholestasis of pregnancy; GDM, gestational diabetes mellitus; HDP, hypertensive disorders of pregnancy; Prenatal steroid, dexamethasone were given within 2 weeks before delivery; SGA, small for gestational age; EOS, early onset sepsis; PDA, patent ductus arteriosus; PS, pulmonary surfactant; msBPD, moderate-to-severe bronchopulmonary dysplasia.

### Screening for predictive factors

Lasso regression was used to select the significant risk factors from the included data. We analyzed 21 risk factors for BPD according to literatures (16, 17). The Lasso regression analysis showed that five perinatal factors were best fit predicting factors for msBPD models: GA, SGA, Apgar 5-min score, EOS, and duration of invasive ventilation.

### Risk prediction nomogram development

**Figure 2:**
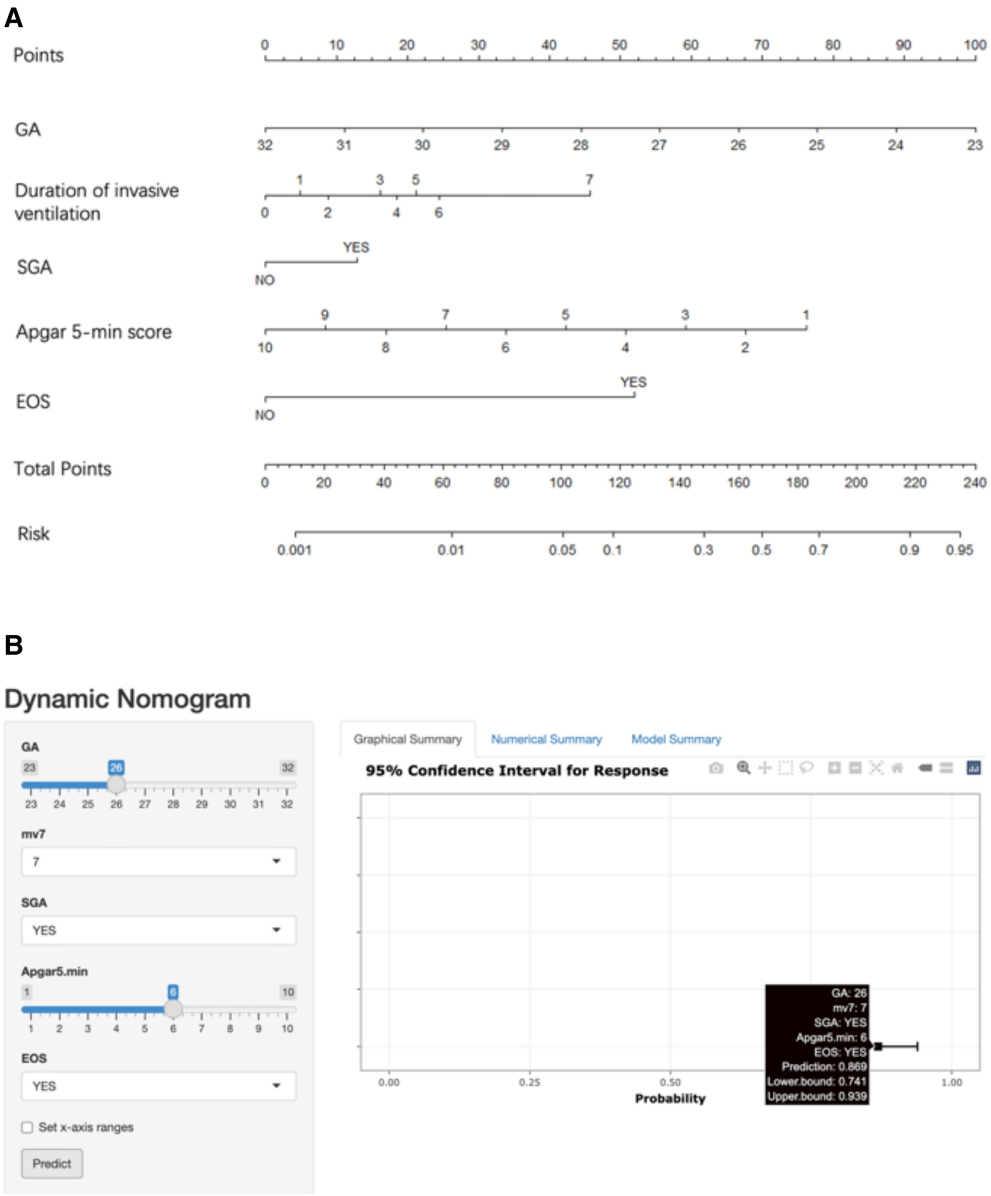
Nomogram. (**A**) Nomogram for the perinatal prediction of msBPD. (**B**) Dynamic nomogram for the perinatal prediction of msBPD. msBPD, moderate-to-severe BPD.

The five factors were integrated into the nomogram [Figure 2A and the available online website: https://sdxxbxzz.shinyapps.io/BPDpredict/ (Figure 2B)]. The dynamic nomogram predicts msBPD within postnatal day 7 based perinatal days. By selecting the corresponding tab, the scores for each variable can be obtained, and the prediction risk associated with the total score represents the risk of developing msBPD. For example, an infant born at 26 weeks, a diagnosis of SGA, Apgar 5-min score of 6, a diagnosis of EOS, and supporting by invasive ventilation for 7 days had the corresponding scores indicating an estimated msBPD of 86.9%. The predicted model had a good fit of the predicted model demonstrated by the Hosmer–Lemeshow test. (*P *= 0.059).

### Predictive accuracy and net benefit of the nomogram

**Figure 3:**
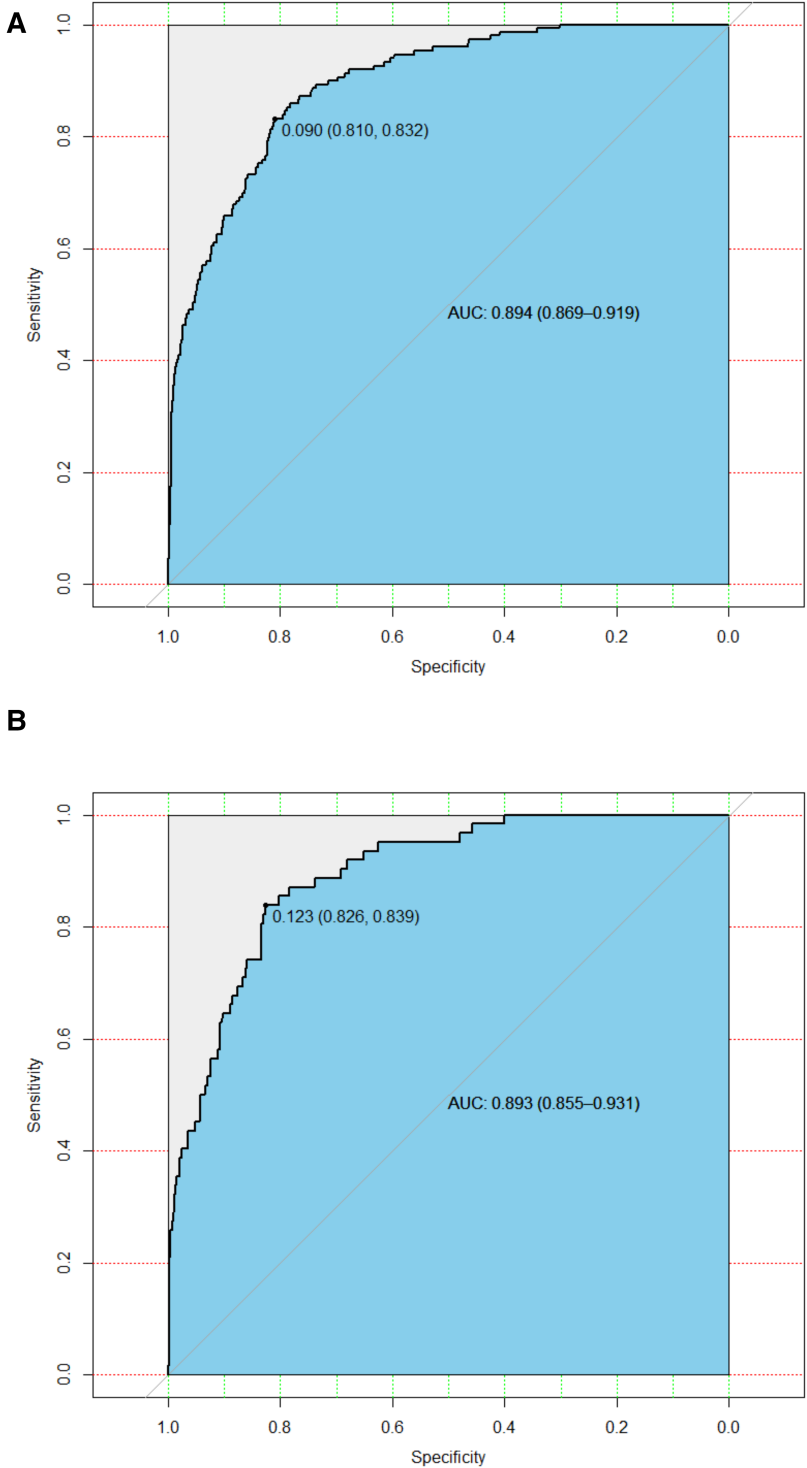
ROC curves. (**A**) Training cohort. (**B**) Validation cohort. ROC, receiver operating characteristic; AUC, area under the ROC curve.

**Figure 4:**
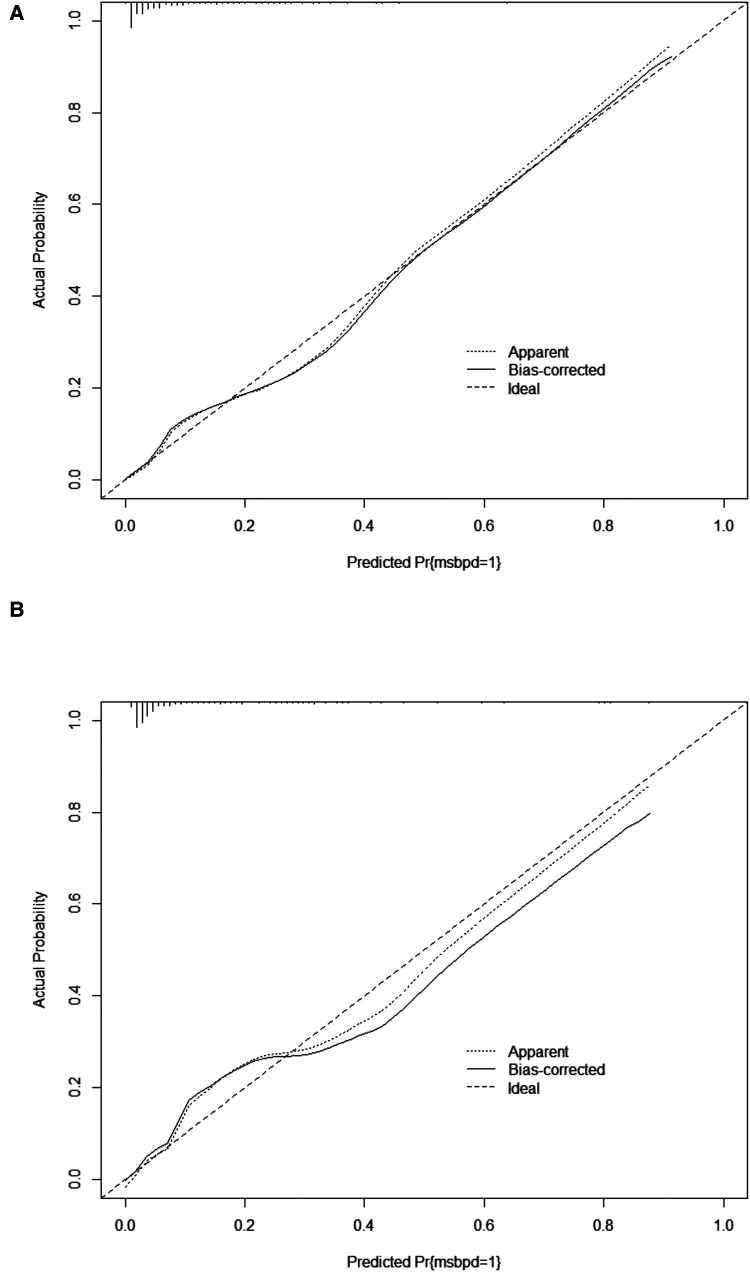
Calibration curve for predicting probability of msBPD. (**A**) Training cohort. (**B**) Validation cohort.

**Figure 5:**
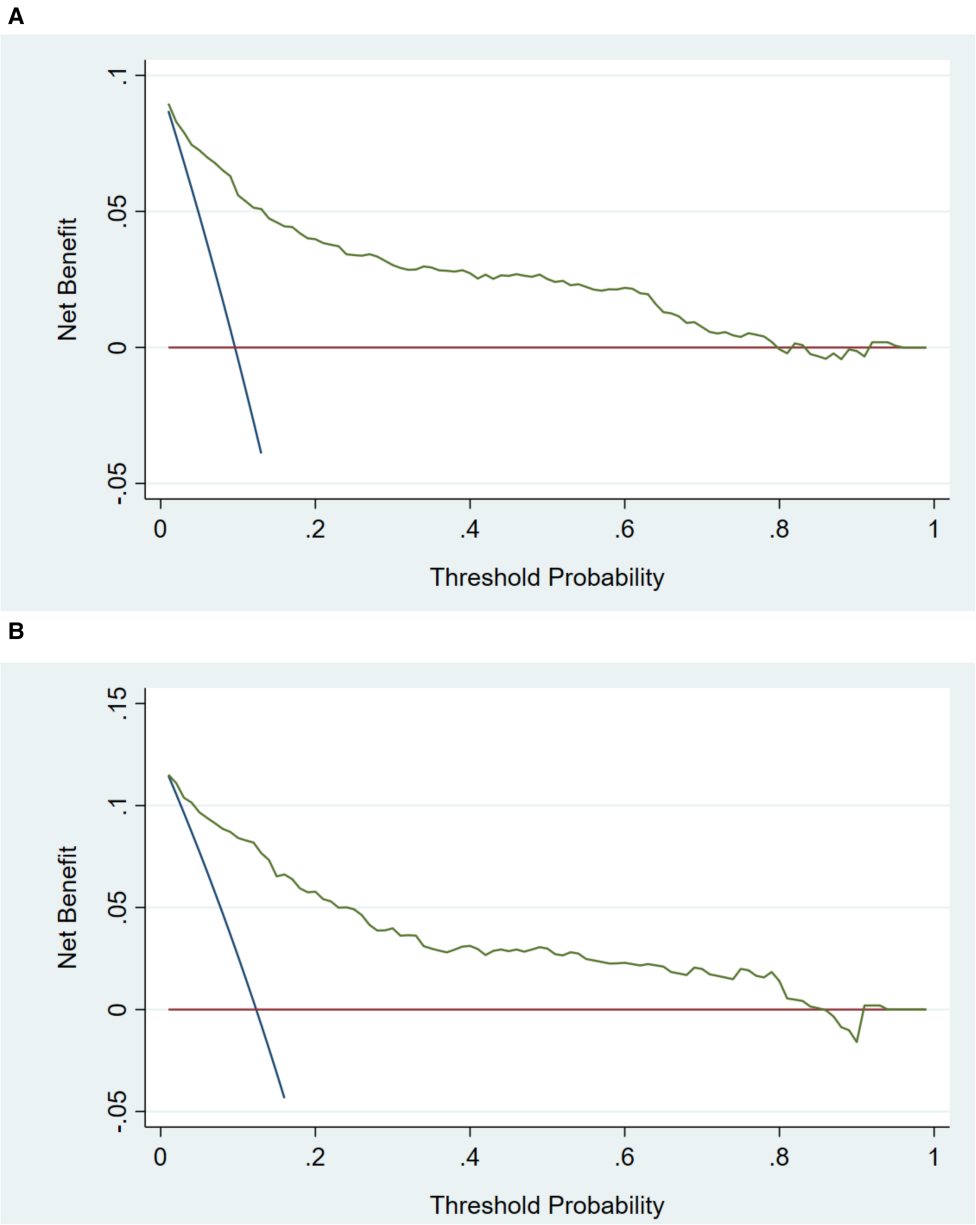
Decision curve analysis in prediction of msBPD. (**A**) Training cohort. (**B**) Validation cohort. The threshold probability for msBPD was indicated by X-axis. And Y-axis indicated the net benefit.

In the training cohort, the AUC and C-index were 0.894 (95% CI 0.869–0.919) (Figure 3A). The calibration curve approach to the ideal diagonal line (Figure 4A) showing high consistencies between the predicted and observed msBPD probability in the cohorts. The net benefit curves for the nomogram in training cohort was shown in Figure 5A. The X-axis indicates the threshold probability for msBPD, while the Y-axis indicates the net benefit. The DCA indicated a significantly better net benefit when the threshold probability was between 0.05 to 0.79, indicating the effective use of the nomogram in achieving net clinical benefit.

In the validation cohort, the AUC and C-index were 0.893 (95% CI 0.855–0.931) (Figure 3B). It demonstrated good predictive ability and accuracy of the nomogram. The calibration curve approached to the ideal diagonal line (Figure 4B), indicating that the prediction model had high goodness of fit. The DCA validation also showed a significant net benefit of the model (Figure 5B).

## Discussion

Our study is the first to develop a dynamic nomogram for predicting the occurrence of msBPD using perinatal factors on a multicenter cohort and provide validation. We validated the high specificity, good sensitivity, and consistency of the prediction model. In their analysis of 26 studies, Onland et al. (10) identified that predictive models for BPD lacked calibration. Peng et al. (18) reported on 18 models for predicting BPD or death in preterm born at ≤32 weeks' gestation, most of which had many methodological shortcomings and lacked calibration assessment. To address these issues, our study included validation cohorts and calibration to enhance the predictive value of our model. Hosmer-Lemeshow test and DCA were used to evaluate calibration and clinical applicability. Thus, we aimed to enhance the predictive value of the model. The model developed by Greenberg et al. (8) included European-American multi-center data with GA of 23 + 0/7 weeks to 28 + 6/7 weeks. The model also lacked validation and calibration. To develop a broadly applicable prediction model for neonatologists, we also include preterm with GA < 32 weeks. As preterm with GA of 29 + 0/7 to 31 + 6/7 weeks accounted for 67.8% of preterm born at <32 weeks' gestation and had a BPD incidence with 19.3% in a Chinese national cohort study (3). We aimed to create a prediction model that can be widely used by neonatologists; our model utilized the Hosmer-Lemeshow test to showcase the calibration, making it more suitable for use in clinical practice.

Our study was a multi-center retrospective study aimed to develop a dynamic nomogram for predicting msBPD in preterm with GA < 32 weeks. Candidate predictors were screened by Lasso regression avoiding overfitting. The AUC of our model was higher than web-based calculator reported by R.G. Greenberg et al. (8) and Laughon MM et al. (7). C-index was 0.894 in training cohort showing favorable discrimination by the dynamic nomogram, which was validated in the validation cohort. DCA evaluates the consequences of the decisions made based on a model. DCA curves demonstrating significant net clinical benefit indicated better prediction of nomogram for predicting msBPD.

The dynamic nomogram can be utilized within postnatal day 7 to predict msBPD. Early prediction allows for early intervention/prevention in NICU. Early predicting msBPD will provide the preterm with msBPD for individualized therapy or precautions. For example, the use of postnatal steroids remains controversial. But the infants with highest risk for developing msBPD need postnatal dexamethasone after the first week of life (16, 19). Early predicting msBPD will also provide clinician for evidence of prevention strategies like diuretic, Vitamin A and nutritional strategies.

The dynamic nomogram computes the incidence of msBPD based on GA, SGA, Apgar 5-min score, EOS, and duration of invasive ventilation. GA is known to be strongly associated with the prognosis of preterm infants as the incidence of BPD is inversely related to GA. Our study also showed that GA was an independent predictor of msBPD and was therefore included in the predictive nomogram. The lower the GA, the higher the number of points in the nomogram and the higher the probability of msBPD. BPD is a chronic lung disease secondary to the critical stages of lung morphological development (tubules and vesicles). Prematurity leads to the early termination of alveolar development and a lack of lung surfactants (20); for this reason, premature infants receive medical intervention immediately after birth, which interferes with normal lung development. Even after prophylactic administration of pulmonary surfactant, low tidal volume, and reduced oxygen concentration, alveolarization and pulmonary vascularization in premature infants are still significantly hindered, making GA one of the most relevant factors for BPD (21).

The Apgar score is one of the most intuitive and convenient tools for assessing the clinical status of newborns immediately after birth. A low Apgar score at 5 min may be the first indication of a poor birth outcome. Barzilay et al. (22) reported that the Apgar score at 5 min, combined with GA, birth weight, and prenatal steroids, can be significant prognostic predictors of outcomes in very low birth weight infants. Our study also showed that the Apgar score at 5 min was negatively correlated with nomogram points. This indicates that the lower the Apgar score at 5 min, the higher the risk of developing msBPD. Low Apgar scores are associated with a higher risk of preterm mortality (23). A low Apgar score indicates that the newborn has poor ability to adapt to the extrauterine environment.

EOS is also considered to be related to intrauterine infection/intra-amniotic inflammation (24), which should be treated with antibiotics. However, most intrauterine infections are difficult to detect. The diagnosis of EOS is definitive, indicating an intrauterine or parturient infection. Inflammation is an important mechanism of BPD (25). Infection/inflammation can cause lung damage and remodeling, which can increase the levels of the inflammatory factors interleukin (IL)-1β and IL-6 (26). Systemic inflammation occurs early in neonates and is associated with BPD, particularly msBPD. Inflammatory cytokines were significantly associated with BPD, especially on day 1 (27). Pathogens colonizing the respiratory tract and the use of antibiotics in preterm infants are risk factors for BPD (28). Inflammation caused by EOS leads to alveolar remodeling and fibrosis in preterm infants. Antibiotics alter the gut microbiota of preterm infants, and dysregulation of the intestinal microbiota can affect the pulmonary immune response through the intestinal-lung axis, thus increasing the risk of BPD (29). In this study, the EOS rate was significantly different between the msBPD and non-msBPD groups (*P *< 0·05). EOS was included in the predictive model. As the diagnostic criteria for EOS are clear and the diagnostic indicators are easy to obtain, EOS is more suitable as a factor for early prediction of BPD.

Our study also included SGA as an independent predictor of msBPD in the predictive model. Our results showed that the number of SGA preterm births in the msBPD and non-msBPD groups was significantly different. Jensen et al. conducted a prospective study showing that SGA was associated with an increased risk of BPD among infants with GA < 32 weeks (30). A multicenter cohort study in China also showed that the incidence of BPD in the SGA group was 3. 1-fold higher than that non-SGA group (31). Most SGA preterm infants have intrauterine growth restriction (IUGR). IUGR is also a major risk factor for BPD. In animal models, studies have demonstrated that IUGR decreases alveolarization and abnormal pulmonary vascular development (32, 33). The SGA is easy to obtain and objective. The inclusion of SGA in the prediction model was also consistent with previous findings that SGA is related to the occurrence of msBPD.

Finally, the duration of invasive ventilation was also included in the predictive model for msBPD. Invasive mechanical ventilation is known to be significantly associated with an increased incidence of BPD (7, 34). Hyperoxia and barotrauma from mechanical ventilation can lead to inflammation, which is a significant risk factor for BPD (35). The Spanish Bronchopulmonary Dysplasia Research Group reported that the length of invasive mechanical ventilation is the most important risk factor associated with type 2/3 BPD (17). Our study also indicated that invasive mechanical ventilation was an independent predictor of msBPD. The longer the ventilation time, the higher the probability of msBPD and the higher the number of points in the nomogram.

In this study, we assessed the perinatal predictors of msBPD in preterm infants with GA < 32 weeks and built a dynamic nomogram for early risk prediction. Our validation confirmed the high specificity and accuracy of the prediction model. A dynamic nomogram provides clinicians with an intuitive and simple tool for practical prediction. Additionally, BPD is a chronic lung disease caused by multiple factors, including inappropriate clinical management. Therefore, we chose perinatal factors for BPD prediction to remove the factors of clinical management. However, our study has some limitations. First, some potential predictors, such as C-reactive protein, procalcitonin, or umbilical cord blood gas, were not assessed due to the lack of data. Second, this multicenter study only included some perinatal centers in a province of China; therefore, additional data from other perinatal centers and specialized children's hospitals are warranted for future analysis.

In conclusion, we found that GA, Apgar 5-min score, EOS, SGA, and duration of invasive ventilation were predictors of msBPD in very preterm infants. We built a dynamic nomogram for the early prediction of msBPD. The higher the total number of points in the dynamic nomogram, the higher the risk of msBPD. The intuitive, personalized, and convenient model of perinatal predictors provides clinicians with a visual and personalized tool for the early identification of msBPD, which may be of significance in reducing complication and mortality rates.

## Data Availability

The raw data supporting the conclusions of this article will be made available by the authors, without undue reservation.
